# A protocol for the development and internal validation of a model to predict clinical response to antihistamines in urticaria patients

**DOI:** 10.1371/journal.pone.0239962

**Published:** 2020-10-06

**Authors:** Jorge Sanchez, Margarita Velasquez, Fabian Jaimes

**Affiliations:** 1 Group of Clinical and Experimental Allergy, "IPS University" Clinic, University of Antioquia, Medellín, Colombia; 2 Dermatological Research Center, University of Antioquia, Medellín, Colombia; 3 Internal Medicine Department, “San Vicente” Clinic, University of Antioquia, Medellín, Colombia; BronxCare Health System, Affiliated with Icahn School of Medicine at Mount Sinai, NY, USA, UNITED STATES

## Abstract

Chronic urticaria causes a significant limitation to quality of life. In the literature, various studies can be found that have reviewed several clinical and laboratory markers, but none of these variables alone is sufficient to predict the patient's prognosis. In this study, we present a protocol to develop a prognostic model that can predict the clinical response of urticaria patients to antihistamines. This is a protocol for a bidirectional cohort study. Urticaria data will be routinely collected from a population of patients over 18 years old. A full multivariable logistic regression model will be fitted, following five steps: 1) Selection of predictive variables for the model; 2) Evaluation of the quality of the collected data and control of lost data; 3) Data statistical management; 4) Strategies to select the variables to include at the end of the model; 5) Evaluation of the performance of the different possible models (predictive accuracy) and selection of the best model. The performance and internal validation of the model will be assessed. Some clinical and paraclinical variables will be measured for further exploration.

## Introduction

Chronic spontaneous urticaria (CSU) is a disease characterized by the sudden appearance of hives and/or angioedema that can cause a significant limitation to quality of life, and it can be associated with different comorbidities such as hypothyroidism, depression, and anxiety. Despite the increased knowledge of its pathogenesis, the evolution of this disease in each patient is currently uncertain. Different studies have reviewed several clinical and laboratory markers to predict the evolution of the disease in terms of its duration or clinical treatment response [1–16].

Despite the fact that different variables have been identified, it seems that none of these variables alone is sufficient to adequately predict any of these outcomes. The construction of a model that includes the set of the most relevant variables could improve predictive accuracy.

The first lines of treatment in urticaria are based on the use of antihistamines; Several patients (40% to 60%) have clinical control with this therapy in conventional or higher doses [17, 18], but the rest of the patients need additional therapies such as the use of omalizumab or cyclosporine, which generates an increase in time and resource costs.

In this article, the following research question is posed: From the clinical characteristics and the IgE and IgG autoantibodies against self-proteins such as thyroperoxidase, could a predictive model be constructed to predict the clinical response to antihistamines in patients with urticaria? The creation of this prognostic model could help identify patients who will have a poor response to antihistamines, and define the early initiation of other therapies.

**Fig 1:**
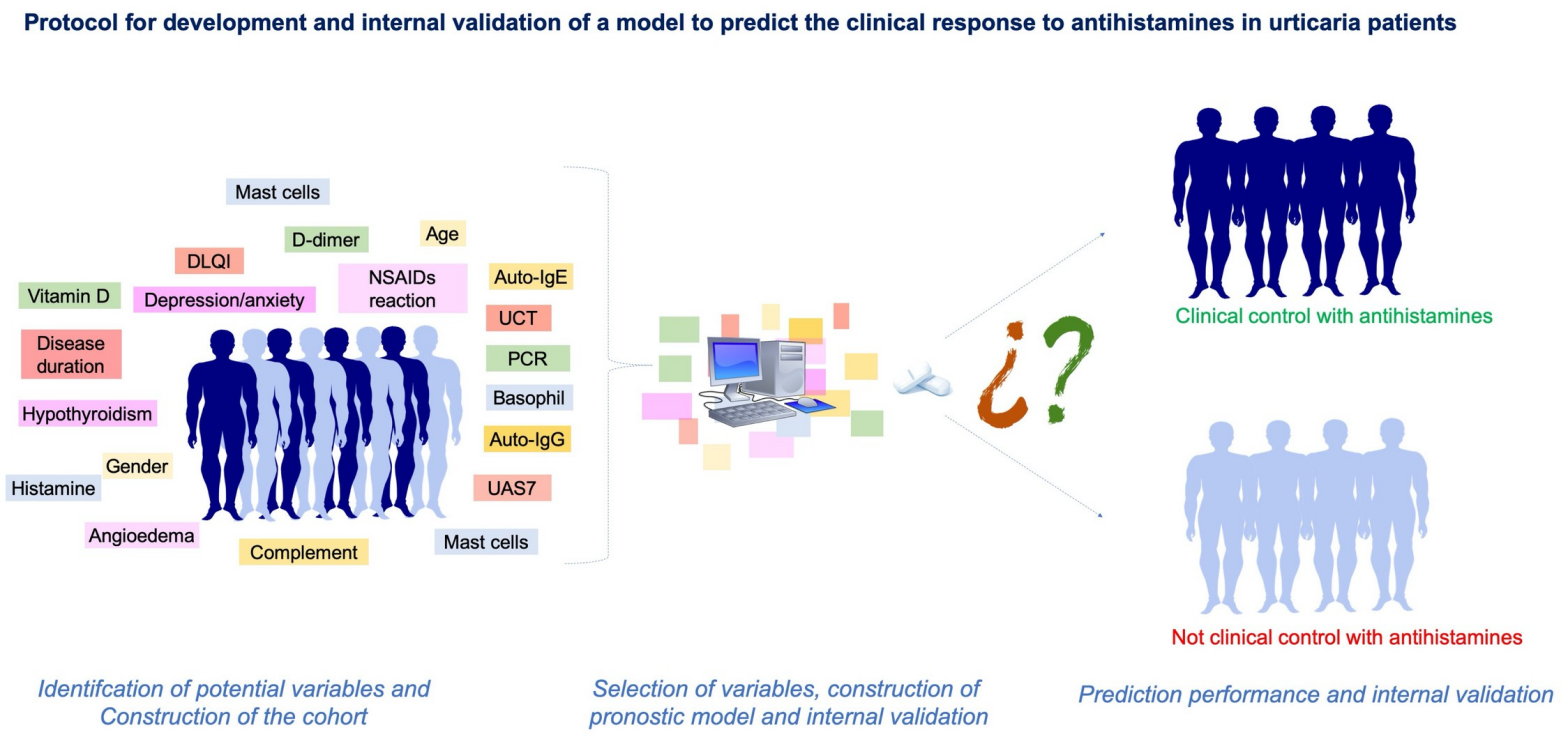
Graphic summary illustrating the steps of the protocol.

Therefore, the main objective of this study is to present a protocol to develop a prognostic model that allows the prediction of clinical response to treatment with antihistamines in adult patients with chronic spontaneous urticaria (Fig 1).

Seeking the best research performance in clinical fields and in favor of collaborative work in the scientific community, this protocol is openly available through this online publication.

## Methods

This is a registered protocol, nevertheless we have ethics committee approved from University of Antioquia (Code F-017-00) and from "IPS Universitaria" clinic (Code 2019–2020).

### Study design and source of data

This is an observational bidirectional cohort study. The first objective will be conducted in accordance with existing guidelines for model development and internal validation [19, 20] and reported in accordance with the “Transparent Reporting of a Multivariable Prediction Model for Individual Prognosis or Diagnosis*”* (TRIPOD) statement [21–24]. Two sources of recruitment will be used from two cities (Medellín and Bogotá) in Colombia:

1) Retrospective data: Patients from the URTICA cohort (URTICA cohort; Urticaria Research of Tropical Impact Control Assessment. Institutional approval IN40-2016, ClinicalTrials.gov identifier: NCT01940393). Those patients who meet the selection criteria for this project will be included if they have all the evaluation variables (predictive variables, outcome variable) and follow-up times.

We will only use retrospective data in case of not reaching a sufficient number of patients in the period of time for the prospective recruitment and in this case comparative analyzes of the retrospective and prospective groups will be made to evaluate the introduction of possible biases.

2) Prospective data: All new patients with a diagnosis of urticaria who attend one of the participating centers and meet the inclusion criteria will be invited to participate.

**Fig 2:**
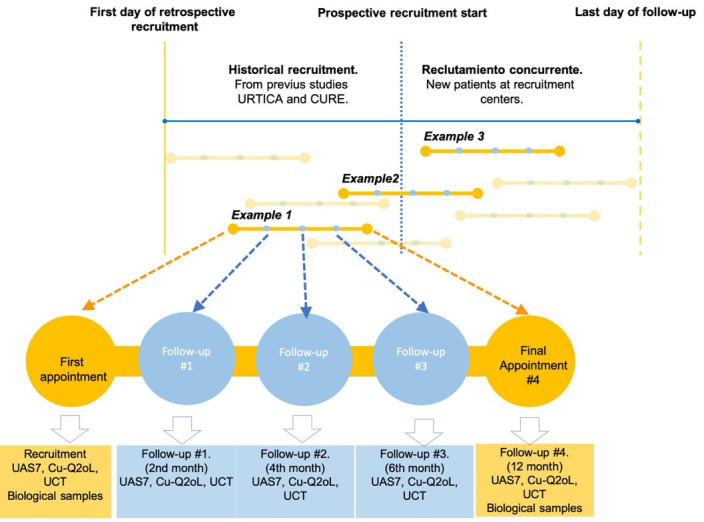
Collection of data. Recruitment will be ambispective. ***Example 1***: Patients from the URTICA [25] and CURE studies (Chronic Urticaria REgistry) who meet the selection criteria, for whom follow-up data are available, and for whom biological samples are available will be included in the study. ***Example 2***: Patients who participate in the URTICA or CURE studies but have not yet been followed up for 2 years can also be included. ***Example 3***: Patients who meet the selection criteria and are prospectively recruited from the participating institutions will be included.

Patients will be recruited from records made from 7 December 2019 to 31 December 2021. Fig 2 shows the different follow-up periods and the procedures performed in each one.

### Participants and eligibility criteria

The reference population will be patients with chronic spontaneous urticaria, over 18 years of age, located in the cities of Medellín and Bogotá (Colombia), and attending dermatology and/or allergy services (secondary care level) at the following institutions: Medellín, Clinic “IPS Universitaria”, Hospital “San Vicente de Paul”, Clinic “Unidad Alergológica”; Bogotá, Clinic “Fundación Santa Fe, IPS UNIMEQ-ORL”. Because these are reference centers in health in the respective cities with hospital and outpatient medical care, the recruited patients will have different levels of severity. CSU medical protocol in these centers is based on the recommendations of international guidelines [27].

The patient inclusion age (≥18 years) is based on the epidemiological peak of the disease [6, 26]. The definition of spontaneous chronic urticaria will be according to the EAACI (European Allergy, Asthma and Clinical Immunology) criteria [27]: the presence of hives and/or angioedema for more than 6 weeks without an explanatory trigger (for example, medications or food). Patients should not have any medical contraindications for the beginning or maintenance of treatment with antihistamines (for example, allergy to the active substance or its excipients, intolerable adverse effects, etc.).

Those patients who suffer from a comorbidity that causes pruritus and could confuse diagnosis will be excluded (for example, dermatitis, chronic itching, scabies, peripheral neuropathy, etc.). Patients who have been treated with monoclonal anti-IgE or any other medication that may affect the evaluation of the clinical impact of antihistamines will not be included [27].

### Outcome and predictors

The outcome variable will be nonresponse to antihistamines in conventional doses or, if necessary, after quadrupling the conventional daily dose for at least one month without obtaining a clinically effective response via the Urticaria Activity Score (UAS7 end <6 points, "control"; UAS7 end >7 points, "no control").

The selection of the model’s prognostic variables was made according to biological plausibility, an exhaustive review of possible variables, and the feasibility of their measurement and reproduction worldwide. We also considered some previous systematic reviews [28–31]. Based on these studies, the variables that we chose to include in the predictive model had different reasons: We include sex and age because they may be related to the outcome of interest but also because they help to control for other confounding factors that may be associated. The duration of the disease, the presence of angioedema, the severity at the onset of symptoms and the presence of comorbidities (inducible urticaria, autoimmunity, NSAIDs, hypersensitivity) seem to be associated with a longer duration of the disease and a lower response to antihistamine treatment.

Anxiety and depression can affect the control of the disease by its association with the severity of the symptoms and the way in which the patient perceives them. States that favor chronic inflammation also appear to be associated with a lower response to antihistamines, so we include variables associated with this state (CRP, body mass index, autoantigens). The predictive variables that we consider evaluating for the model are described in Table 1. In this study, considering the cohort design and that the outcome is easily objectifiable (formation of wheals or not), a blind assessment of the outcome to be predicted is not necessary. All predictors will be measured at the first appointment, prior to the start of continuous treatment with antihistamines.

**Table 1:** Predictive variables to consider in the model.

Variable	Nature	Variable type	Instrument/stand
Age	Continuous (years)	Predictive	Identification
Sex	Dichotomous (Yes/No)	Predictive	Identification
Urticaria time	Continuous (Months)	Predictive	Self-report
Angioedema	Dichotomous (Yes/No)	Predictive	Self-report/Photo
Inducible urticaria	Dichotomous (Yes/No)	Predictive	Self-report/Challenge test
UAS7 baseline	Continuous	Predictive	UAS7 scale baseline
Autoimmunity	Dichotomous (Yes/No)	Predictive	Dx autoimmune diseases and paraclinical test
Anxiety and/or depression	Dichotomous (Yes/No)	Predictive	Goldberg scale
Atopy	Dichotomous (Yes/No)	Predictive	SPT and/or sIgE
IgE TPO	Continuous (Unit/ml)	Predictive	Paraclinical test
IgG TPO	Continuous (Unit/ml)	Predictive	Paraclinical test
Eosinophils	Continuous (count/ml)	Predictive	Paraclinical test
C reactive protein	Continuous (count/ml)	Predictive	Paraclinical test
AINEs hypersensitivity	Dichotomous (Yes/No)	Predictive	Self-report/Challenge test
Body mass index	Continuous	Predictive	Height and weight
UAS7 final	Continuous	Outcome	UAS7 scale final*

As the main outcome, clinical control (UAS7 control <6 points or no UAS7 control >7) will be determined after at least four weeks with the conventional dose and increased dose of an antihistamine.

### Sample size

Nonprobability convenience sampling will be carried out according to the order of arrival at clinical centers. The sample calculation will be according to the criterion of "events by variable" for predictive indices [32, 33]. This criterion is based on obtaining a number of outcomes for each predictive variable studied. Considering categorical variables that may require a greater number of patients to be evaluated and the frequency of the prognostic variables to be included in the model, we decided to include 20 subjects (potential outcomes) for each variable. Considering that 30 to 70% of patients achieve symptom control with antihistamines according to UAS7 (outcome of interest), and based on a previous study in the cities where the recruitment will take place [17], we consider that this outcome could occur in 50% of our population, so the formula applied for sample size would be the following:

n = 20*k/i where "k" is the number of predictive variables and "i" is the incidence of the outcome of interest: n = 20*15/0.5 = 600. Expecting a loss of 10% (60 patients), the minimal sample size would be 660 patients.

### Missing data

The multiple imputation strategy will be applied to complete the possible missing data that we obtain from the variables as long as they do not exceed 10% of the data and the lost data can be assumed to be by random causality (when the probability that the data are lost is independent of the observed and unobserved characteristics of the sample); otherwise, if lost data of a variable exceed 10% or are not random, the variable should be removed from the model.

For this study we will use the interactive Monte Carlo and Markov chains (MCMC) method, since it provides ease of use when the loss pattern does not have characteristics of monotonic function.

### Statistical analysis methods

For the analysis and construction of the model, once the information has been collected, missing or atypical data will be verified to avoid inconsistencies, and a descriptive analysis of the characteristics of the patients admitted to the institutions of interest will be carried out. The mean, standard deviation, and range will be used as summary measures when it comes to quantitative variables. For qualitative variables, frequency distributions and percentages will be used.

**Fig 3:**
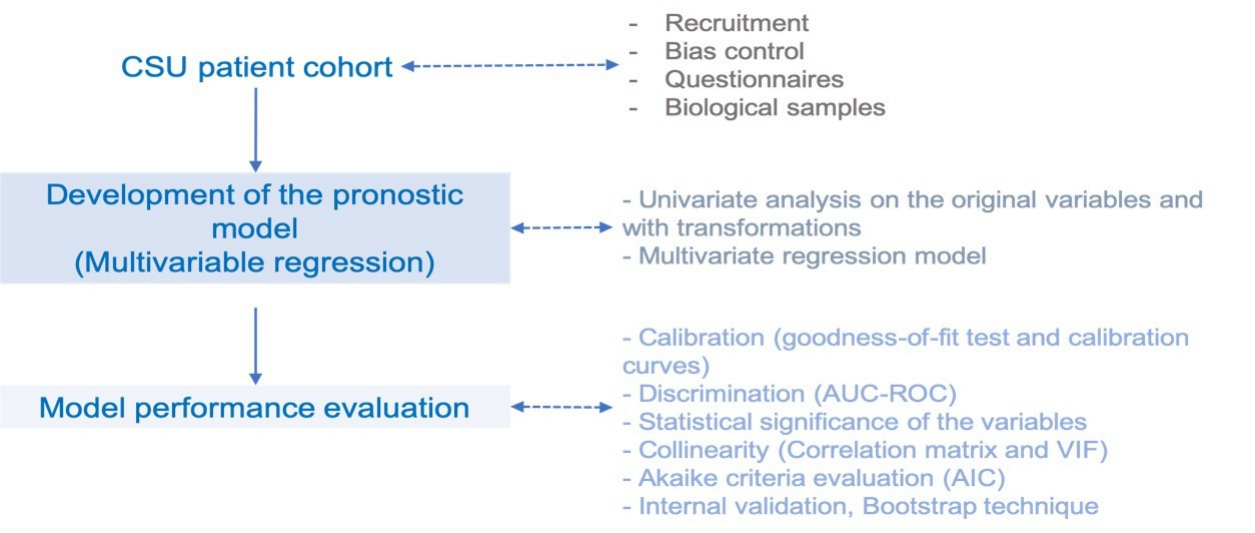
Methodological summary. Summary of the model according to the methodology used in the three main steps: 1) construction of the cohort, 2) development of the prognostic model, and 3) evaluation of model performance.

There are five steps to follow in the processing of the variables and the analysis of the data (Steps 1, 2, and 3 are the results of decisions made prior to the construction of the model) (Fig 3):

#### Step 1: Selection of variables for the model

The selection of the model’s prognostic variables was made according to biological plausibility, an exhaustive review of the variables that have been previously studied [12, 15, 28, 30, 31, 34–38], and the feasibility of their measurement in clinical practice (Table 1).

#### Step 2: Evaluation of the quality of collected data and management of lost data

The steps to follow in case of requiring imputation will be the following:

1. Summary of missing values via graph and the percentage of missing data.

2. Patterns of missing values: The tabulation of missing data can indicate whether the losses follow a monotonic pattern and the type of imputation should be changed.

3. Generation of five different imputations.

4. Weighting of multiple imputations to obtain a single database.

#### Step 3: Data management

Analysis of the collinearity assumption in continuous variables will be applied by means of the correlation matrix (where a value of r > 0.7 is considered to indicate collinear variables) or by the variance inflation factor (VIF > 5 indicating collinearity) and in categorical variables by means of the chi-square test of independence (relationship between categorical variables with p > 0.05 indicating collinearity).

The monotonic function relationship assumption will be evaluated in the continuous variables graphically using the Lowess function; those variables that by graphic criteria do not meet this assumption will be transformed or dichotomized.

#### Step 4: Strategies to select the variables to include at the end of the model

For each of the variables that remain in the model, the degree of association with the dependent (outcome) variable (bivariate analysis) will be estimated by calculating the odds ratio (with its corresponding 95% confidence interval), and a multivariate regression model will be applied. To select the independent variables associated with the outcome of interest, the Wald statistic will be evaluated, considering p < 0.05 to include a variable as significant in the multivariate model and/or biological plausibility.

#### Step 5: Evaluation of model performance (predictive accuracy) and internal validation

To compare the predictive capacity of the models, we will evaluate the discrimination and calibration: *Discrimination* will be evaluated in each of the models graphically and by using the c statistic and area under the ROC curve (Receiver Operating Characteristic). *Calibration* tells us about the goodness of fit of the model; we will use the Hosmer–Lemeshow hypothesis test (a value of p > 0.05 will indicate that the model has good calibration).

For the selection of the best model, several models will be built according to the inclusion and exclusion of variables. To compare these prognostic models, the data obtained from a bootstrap analysis will be used as a comparison sample (internal validation). Among the models, we will evaluate the discrimination improvement index, IDI (Integrated Discrimination Improvement), and the reclassification improvement index, NRI (Net Reclassification Improvement) [39].

The IDI linearly evaluates the change in the probability estimate of the outcome between the models. It will be calculated as the difference between the means of the estimated probabilities of the outcome of one model versus those estimated in the other, minus the same difference between those who did not have the outcome. In other words, the IDI represents the average improvement of a model in terms of the prediction of patients who actually have the outcome, removing what is worsened by the prediction of the result in patients who ultimately do not have it. The NRI considers changes between the different risk categories (success and nonsuccess). To estimate the categorical NRI, the outcome will be used and two comparison tables will be constructed separately according to whether or not the event of interest has been recorded in the patients.

The Akaike information criterion (AIC) and Bayesian information criterion (BIC) will also be evaluated (the lowest value obtained will define the best model) for the selection.

In case the final model has good performance, the specifications of the model, its limitations, and its interpretation will be explained in detail. The analyses described will be run using R, SPSS, and/or STATA software.

### Model score and presentation format

The model score is closely tied to the presentation format. Ideally, we want to make a model with variables in their natural form (not transformed) that allows us to establish a risk score and offer it in the form of an electronic application available on computers and cell phones. In this way, the clinical prediction model provides an estimate of risk with a wide range (relatively low to relatively high risk), according to patient characteristics easy to use for the physician.

However, during the development of the model it may be necessary to carry out some transformations and according to the performance of the predictive model and the feasibility of its use for the clinician, we can consider dichotomizing the result variable and using other forms of presentation like example model, spreadsheet or score chart.

### Limitations

The cohort design, realization by trained personnel, monthly interim analysis, and joint training of the different centers with the periodical monitoring that we plan to carry out will help to reduce selection, measurement, and confusion biases [40]. We can detect some limitations: the selection criteria and sampling of the retrospective cohort could result in differences with the prospective cohort; however, because the population, recruitment, and selection protocol are the same in the two studies and the time difference between the patients selected in the retrospective and prospective cohorts is less than one year, we consider that these risks are minimized.

Patients with CSU may have variable intensity of symptoms, and those with weaker symptoms are likely to have less motivation to participate. Although this is a problem that may affect the generalizability of the results and the applicability of the model in patients with mild symptoms, it has equal influence on the groups that result for the outcome of interest (responding or not to antihistamines), which allows to avoid imbalances in the interpretation.

The measurement of the laboratory variables will be carried out in duplicate; additionally, the measurements will be carried out in two laboratories, which will reduce the risk of error in the measurement of these variables.

Considering that the outcome of the study is to evaluate the clinical control achieved with antihistamines, it is possible that the group of subjects who achieve rapid control will be more tempted to leave the study than the group that does not obtain an adequate response. To control this bias, the importance of continuing in the study until the end of the same will be emphasized to the study participants at the time of recruitment; they will also be asked to agree to receive calls or visits, which will remind them of follow-up appointments and/or data collection.

Due to the fact that clinical control can be temporary, patients will be followed up over time (Fig 2) for a minimum of three months to define whether control is constant over time.

## Ethical considerations

The investigation will be carried out in each center when authorization is obtained from the legal representative of each institution and the respective ethics committee. Each research subject will provide a written informed consent. In the event that a methodological change is required during the execution of the project, the amendments will be submitted again to the ethics committee.

In case of not obtaining the necessary number of patients in the recruitment period, we will use retrospective data from two previous projects that are in development and that have been approved by the institutional ethics committees. In these previous urticaria studies, each patient was asked for a written informed consent, where they authorized the use of the data for related projects with prior authorization from the ethics committee.

In this project, we will comply with the current national regulations on research and international regulations; we adhere to the recommendations of the Nuremberg code (1946), the UNESCO Universal Declaration on Bioethics and Human Rights (2005), and the Helsinki declaration (2013), and we will follow international ethical guidelines for health-related research (CIOMS 2018) in the aspects that correspond to this project.

## Implications of this protocol

Although several variables that seem to be associated with the clinical response to antihistamines in CSU patients have been identified, at the moment, none is sufficient to make a correct prediction. Multivariable models could be a method to achieve through a set of variables what cannot be achieved through them separately. No multivariate models have been developed or validated to predict the response to antihistamines in CSU patients; our research has this objective and could benefit patients from different populations since this prediction model would allow for identifying those patients in whom antihistamine therapy is adequate and those patients who would require other therapies, reducing the use of unnecessary management. Conventional doses of antihistamines and increased dosages are the first two management steps according to current urticaria guidelines, and patients should wait up to four weeks to go from one treatment stage to another. In the patients who will fail to respond to the antihistamine, the predictive model would allow faster treatment adjustments, leading to better quality of life when controlling the disease, while on the other hand allowing better use of medical appointment resources, emergency consultations, and drug costs.

This research may also offer valuable insights into practical aspects of the clinical and molecular characteristics of urticaria since it allows for identifying possible endophenotypes of patients according to the clinical and laboratory characteristics associated with response (or lack thereof) to antihistamines.

The variables chosen as candidates for the predictive model come from several previous studies, some of which have been replicated in different populations and have biological plausibility, which makes them good candidates for the outcome of interest. The prospective recruitment of the study allows us to include the a priori candidate variables that we believe are most relevant as long as we meet the pre-established sample size defined by the ratio of number of events per variable, to avoid overestimation of the model.

According to the performance of the final model, it may be possible to evaluate reproducibility and transportability [19, 41]. This predictive model is intended to be applied in a population with CSU that is going to formally start its management with antihistamines. Since the model is initially being developed for use by dermatologists and allergists, it will later be necessary to evaluate its use by general practitioners.

Among the selected variables, some correspond to clinical and molecular variables; however, most of them are nonmodifiable. Therefore, in terms of prevention strategies, they have limited utility, but this does not affect the predictive capacity of the model and could facilitate the external reproduction process since they are not variables that are modified by environmental factors.

We hope that this study will also identify further research priorities for this novel and potentially valuable area of urticaria research; further hypothesis-free exploratory studies that investigate many factors may assist with the identification of new factors (biologically associated or not) that may help in management decisions and for monitoring purposes [42]. Furthermore, these types of studies may be used to update and improve the performance of the predictive model [43].

Finally, once the final results of the study will be published, seeking the greatest possible transparency, the source of the analysis database will be attached to the article but recognizing the possible restrictions that the participating centers and journal has in this regard (e.g., extension of supplementary material, type of authorized material, preserve the privacy of each patient).

In conclusion, we have developed a protocol for creating a multivariable prognostic model focused on predicting the response to antihistamines in patients with CSU. From this model, new lines of research can emerge and contribute both to the external validation of the model and to the construction of new models with different outcomes.
